# The potential economic burden of Zika in the continental United States

**DOI:** 10.1371/journal.pntd.0005531

**Published:** 2017-04-27

**Authors:** Bruce Y. Lee, Jorge A. Alfaro-Murillo, Alyssa S. Parpia, Lindsey Asti, Patrick T. Wedlock, Peter J. Hotez, Alison P. Galvani

**Affiliations:** 1 Public Health Computational and Operations Research (PHICOR) and Global Obesity Prevention Center (GOPC), Johns Hopkins Bloomberg School of Public Health, Baltimore, Maryland, United States of America; 2 Center for Infectious Disease Modeling and Analysis, Yale School of Public Health, Yale University, New Haven, Connecticut, United States of America; 3 Sabin Vaccine Institute and Texas Children’s Hospital Center for Vaccine Development, National School of Tropical Medicine, Baylor College of Medicine, Houston, Texas, United States of America; Colorado State University, UNITED STATES

## Abstract

**Background:**

As the Zika virus epidemic continues to spread internationally, countries such as the United States must determine how much to invest in prevention, control, and response. Fundamental to these decisions is quantifying the potential economic burden of Zika under different scenarios.

**Methodology/Principle findings:**

To inform such decision making, our team developed a computational model to forecast the potential economic burden of Zika across six states in the US (Alabama, Florida, Georgia, Louisiana, Mississippi, and Texas) which are at greatest risk of Zika emergence, under a wide range of attack rates, scenarios and circumstances. In order to accommodate a wide range of possibilities, different scenarios explored the effects of varying the attack rate from 0.01% to 10%. Across the six states, an attack rate of 0.01% is estimated to cost $183.4 million to society ($117.1 million in direct medical costs and $66.3 million in productivity losses), 0.025% would result in $198.6 million ($119.4 million and $79.2 million), 0.10% would result in $274.6 million ($130.8 million and $143.8 million) and 1% would result in $1.2 billion ($268.0 million and $919.2 million).

**Conclusions:**

Our model and study show how direct medical costs, Medicaid costs, productivity losses, and total costs to society may vary with different attack rates across the six states and the circumstances at which they may exceed certain thresholds (e.g., Zika prevention and control funding allocations that are being debated by the US government). A Zika attack rate of 0.3% across the six states at greatest risk of Zika infection, would result in total costs that exceed $0.5 billion, an attack rate of 1% would exceed $1 billion, and an attack rate of 2% would exceed $2 billion.

## Author summary

Our work forecasts the cost and economic burden of the Zika virus across six states in the US which are at the greatest risk of Zika (Alabama, Florida, Georgia, Louisiana, Mississippi, and Texas). We evaluated the burden by varying attack rates, scenarios, and circumstances. All costs are reported in 2016 dollars. This is the first study to determine the potential economic impact of the Zika epidemic in the US across a wide range of possible scenarios (e.g., different scenarios explored the effects of varying attack rates from 0.01% to 10%). There has previously been debate regarding resource allocations to address the Zika epidemic. Our results are important for decision makers because they identify thresholds at which the economic impact of Zika on these six US states would exceed $0.5, $1.0, $1.5, and $2.0 billion. For example, a Zika attack rate of 1% across Alabama, Florida, Georgia, Louisiana, Mississippi, and Texas, the six states at greatest risk of Zika infection, would result in total costs that exceed $1 billion. Our study also reveals the primary determinants of Zika-associated costs, which can help guide future data collection and control efforts.

## Introduction

As the Zika virus epidemic continues to spread internationally, countries such as the United States must determine how much to invest in prevention, control, and response. Fundamental to these decisions is an understanding of the potential economic burden of Zika. For example, former US President Barack Obama proposed allocating $1.8 billion towards Zika response programs, which included $828 million distributed towards the Centers for Disease Control and Prevention (CDC) and $210 million towards the US Department of Health and Human Services Response Activities, the majority of which was proposed to be allocated to address Zika on the continental US.[1] US Congress passed a bill that allocates $1.1 billion for Zika response and preparedness.[1] Critics of the initial $1.8 billion funding request have claimed that the former President’s proposed budget allocation is excessive, while others have countered that such an investment is appropriate given the current extent of the outbreak and potential for further devastation.[2] However, without quantification of the possible economic burden of the outbreak, both sides of the debate have had limited data on which to base their arguments.

Evaluating the potential impact of the Zika epidemic can be difficult without the aid of computational approaches. Zika infection can lead to a constellation of symptoms that include fever, rash, joint pain, conjunctivitis, muscle pain, and headache[3] that have myriad repercussions on medical costs and productivity. The Zika virus has also been linked to more substantial clinical sequelae, such as Guillain-Barré Syndrome (GBS),[4, 5] and severe birth defects, including microcephaly.[6, 7] Therefore, we developed a computational model to inform decision making and forecast the potential economic burden of Zika in six at-risk US states under a wide range of different hypothetical scenarios and circumstances.

## Methods

### Model structure

**Fig 1:**
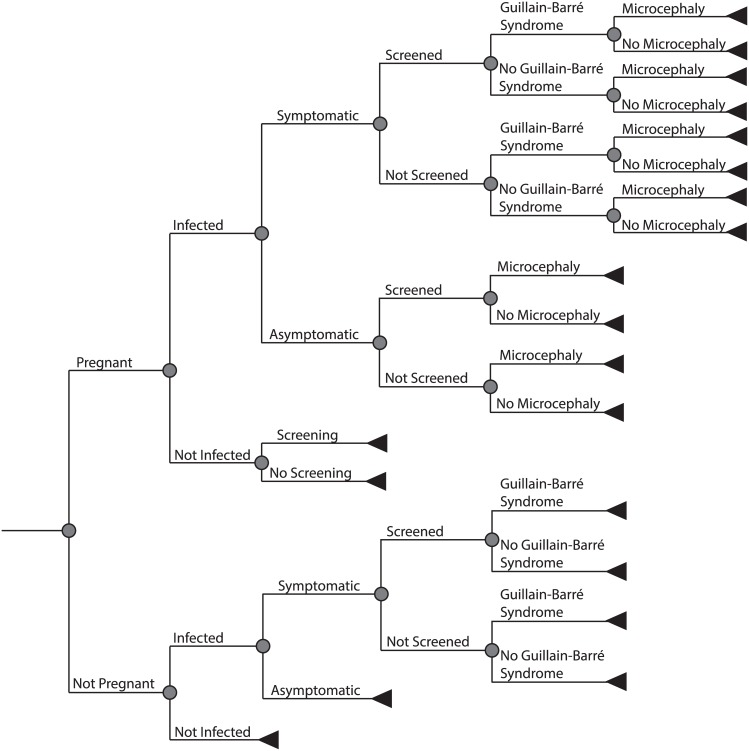
Model structure. The flow diagram above illustrates each of the paths an individual in the model can take in the event of a Zika virus outbreak, given different probabilities at each node. The model includes all individuals in the six selected states considered at risk for Zika virus infection in the US.

Through substantial adaptation and extension of our previously published model,[8] we developed an economic model to determine Zika-related costs in six states in the United States under a variety of attack rate scenarios (Fig 1). We varied the risk of Zika virus infection among all individuals living in one of the six states by attack rate and stratified the populations by pregnancy status to estimate the risk of Zika-related birth defects. In our model, each non-pregnant Zika case has a probability of being symptomatic or asymptomatic. We utilized the cost of care for conjunctivitis as a proxy for clinical costs for symptomatic Zika, as conjunctivitis is a common clinical symptom of Zika infection[4] and a clear clinical course.[3] We varied this cost by scenario: 1) an outpatient primary care visit (conservative scenario), 2) a specialist visit (base case), and 3) both a primary care and specialist visit (less conservative scenario).[9] Symptomatic Zika cases also have a probability of developing GBS.

Pregnant women can be infected or uninfected. Based on CDC guidelines,[10] if uninfected, a pregnant woman is still considered at risk for Zika infection and can potentially visit a clinic to have an ultrasound and reverse transcription-polymerase chain reaction (RT-PCR) test performed. If infected, pregnant women have a probability of being symptomatic or asymptomatic. Both symptomatic and asymptomatic infected pregnant women can be screened for Zika. Asymptomatic, Zika-infected pregnant women undergo an additional ultrasound and are screened using real-time RT-PCR (rRT-PCR) on serum and urine. We conservatively assumed that the rRT-PCR tests on serum and urine are the only tests an asymptomatic pregnant woman will undergo, although CDC guidelines for asymptomatic Zika-infected pregnant women can include a Zika virus immunoglobulin M (IgM), a dengue virus IgM (used to rule out dengue infection), and a plaque reduction neutralization test (PRNT) depending on initial test results. Symptomatic, infected pregnant women undergo screening for Zika using all the aforementioned methods (rRT-PCR and PRNT) as well as Zika virus IgM and dengue.[10] Each Zika-infected pregnant woman has a risk of GBS and a probability of delivering an infant born with microcephaly and other severe central nervous system (CNS) disorders. Pregnant, infected women with abnormal results, either by ultrasound or serology testing, require additional screening, which includes serological testing for other infections, a series of follow-up ultrasounds, and potential amniocentesis. For each potential hospital visit (e.g., outpatient, specialist, screenings, ultrasounds), different scenarios evaluated the effects of varying compliance, from 30% to 90%, a range which included values reported in the literature (85.8% and 73.7% [11, 12]). The frequency of the tests used for the different at risk populations can be found in Table 1.

**Table 1:** Key model inputs.

Parameter	Base case	More conservative	Less conservative	Source
**Probabilities (%)**				
Symptoms given Zika infection	18.36	9.59	27.11	[3]
Infant with Microcephaly or other central nervous system (CNS) disorders when pregnant mother infected with Zika	3.43	0.95	19.05	[18, 19]
Guillain-Barré Syndrome (GBS) when infected with Zika	0.06	0.02	0.08	[8]
Compliance/adoption factor of treatment guidelines	60	30	90	[11, 12]
Patient is on Medicaid				
Alabama	22	—	—	[20]
Florida	20	—	—	[20]
Georgia	18	—	—	[20]
Louisiana	31	—	—	[20]
Mississippi	25	—	—	[20]
Texas	16	—	—	[20]
**Costs (US$ 2016)**				
Microcephaly and other CNS disorders				
Direct medical cost per case	593,522	—	—	[14, 16]
Productivity losses per case	3,516,025	—	—	[14, 16]
Total lifetime cost per case	4,109,547	—	—	[14, 16]
Guillain-Barré Syndrome				
Direct medical cost per case	57,107.56	47,223.00	66,992.12	[15]
Productivity losses per case	344,988.85	303,707.17	386,270.53	[15]
Total lifetime cost per case	402,096.41	350,930.17	453,262.53	[15]
Zika virus (at-risk and/or infected, non-GBS/non-CNS costs)				
Outpatient Visit	—	51.52	51.52	[21]
Specialist Visit	100.97	—	100.97	[21, 22]
Screening visit	100.97	51.52	152.49	[21, 22]
Dengue virus immunoglobulin M (IgM)	12.67	12.67	12.67	[21, 22]
Zika virus IgM	12.67	12.67	12.67	[21, 22]
Plaque Reduction Neutralization Test (PRNT)	21.77	21.77	21.77	[21, 22]
Reverse transcription-polymerase chain reaction (RT-PCR)	35.00	35.00	35.00	
Ultrasound visit	100.97	51.52	152.49	[21, 22]
Ultrasound test	92.73	92.73	124.24	[21, 22]
Broad serological screening	107.60	107.60	107.60	[21, 22]
Amniocentesis	226.27	226.27	226.27	[21, 22]
Hourly Wage	20.58	20.58	20.58	[13]
**Counts**				
Symptomatic Zika infection, non-pregnant, non-complicated				
Outpatient Visit	—	1	1	[23]
Specialist Visit	1	—	1	[23]
Pregnant, uninfected				
Screening visit	1	1	1	[23]
RT-PCR	1	1	1	[10]
Ultrasound visits (1 test per visit)	1	1	1	[23]
Pregnant, Zika-infected, normal ultrasound				
Screening visit	1	1	1	[23]
Dengue virus IgM**	1	1	1	[10]
Zika virus IgM**	1	1	1	[10]
RT-PCR*	2	2	2	[10]
Ultrasound visits (1 test per visit)	3	1	3	[23]
Pregnant, Zika-infected, abnormal ultrasound				
Screening visit	1	1	1	[23]
Dengue virus IgM	1	1	1	[10]
Zika virus IgM	1	1	1	[10]
PRNT	1	1	1	[10]
RT-PCR*	2	2	2	[10]
Ultrasound visits (1 test per visit)	3	3	5	[23]
Broad serological screening	1	1	1	[23]
Amniocentesis	1	1	1	[23]
**Duration (hours)**				
Time missed from work per clinic visit	4	2	8	[24, 25]
**Other**				
Population Estimate (2015)				
Alabama	4,858,979	—	—	[26]
Florida	20,271,272	—	—	[26]
Georgia	10,214,860	—	—	[26]
Louisiana	4,670,724	—	—	[26]
Mississippi	2,992,333	—	—	[26]
Texas	27,469,114	—	—	[26]
Birth Rate (number births/total population)				
Alabama	1.23	—	—	[27]
Florida	1.11	—	—	[27]
Georgia	1.30	—	—	[27]
Louisiana	1.38	—	—	[27]
Mississippi	1.29	—	—	[27]
Texas	1.48	—	—	[27]

* Used dengue IgM as proxy

** Conservatively used only when cases were symptomatic

### Costs

We estimated costs from the third-party payer perspective (i.e., direct medical costs) and societal perspective (i.e., total costs; the sum of direct medical costs and productivity losses). In addition, we calculated the proportion of direct medical costs that would be covered by Medicaid. Direct medical costs include the compliance-adjusted costs for all hospital visits and medical procedures for Zika-infected pregnant and non-pregnant persons, non-infected pregnant women, microcephaly and other CNS disorders in neonates, and GBS cases.

Productivity losses resulted from both healthcare visits and time missed due to illness, with median national wage per hour serving as a proxy for the rate of productivity losses.[13] For the societal perspective, time lost was valued equally regardless of employment status. A healthcare outpatient visit resulted in 2 to 8 hours of productivity losses, in our more and less conservative scenarios, respectively (Table 1). Estimates of productivity losses from microcephaly and other CNS disorders came from previous estimates for autism,[14] and another study was used to estimate the productivity losses due to GBS.[15] The overall consumer price index [16] adjusted all past costs to 2016 US dollars. In order to capture contributions to society that may occur beyond standard employment (e.g., those in school who become ill would incorporate caregiver productivity losess), our base case scenario incorporated productivity losses from those formally employed and unemployed. Based on the lifetime total cost and direct medical cost per person with autism provided by Ganz et al.[14], we were able to calculate lifetime total cost and direct medical cost in 2016 US dollars, $4,109,547 and $593,522 respectively, and apply it to those with microcephaly (Table 1). We were able to do this using the lifetime total cost and direct medical cost for GBS estimated by Frenzen et al.[15] ($350,930 and $47,223, respectively).

Since productivity losses are a substantial piece of the total economic burden associated with a Zika outbreak, sensitivity around these costs are important. As a conservative analysis, we confined productivity losses to those of employable age (16+). We used state-level employment statistics to determine the percentage of the working-age population employed either full-time or part-time in the civilian labor force.[17] We converted this to capture the percentage employed across the entire population of each state and used this percentage to recalculate productivity losses.

### Data sources

Table 1 shows the inputs for our model, divided into probabilities, costs, durations, and other types of inputs. Studies of the ongoing outbreak in Rio de Janeiro, Brazil yielded the base case probability of an infant being born with microcephaly and other CNS disorders[18] as well as our less conservative estimate for the percentage of CNS cases per pregnant women infected with Zika.[18] Results from the 2013–2014 French Polynesia Zika outbreak were used for the more conservative probability of microcephaly.[19] We used the probability of Zika-linked GBS from our previously published model with data from Brazil and Colombia, namely 0.2, 0.6, and 0.8 per 1000 population infected for the more conservative, base case, and less conservative scenarios, respectively.[8] We used the Clinical Laboratory Fee Schedule from the Centers for Medicare & Medicaid Services (CMS) for costs of the laboratory testing (Table 1).

### Scenarios

This study consisted of running various hypothetical scenarios to explore the impact of varying attack rates (from 0% upwards) in each of the six states (Alabama, Florida, Georgia, Louisiana, Mississippi, and Texas). We focused on the Gulf Coast states and Georgia given their suitability of environmental conditions to the *Aedes aegypti* and *Aedes albopictus* mosquito vectors [28, 29] and their relative proximity to the ongoing outbreak in Latin America, including Puerto Rico. Monaghan et al. shows, the potential range of both these vectors covers most, if not all of the state.[29] It is unknown what the true attack rate will be in these states, thus we provide a large range to show how the burden varies under different attack rate scenarios. While the attack rate for Zika may be similar to that of dengue, as they are transmitted from mosquito bites from the same species, it also may be higher as Zika can be transmitted through sexual contact.[30] Additional sensitivity analyses assessed the impact of varying key model parameters across the ranges parameterized from clinical, epidemiological, and cost data (Table 1). For example, we varied the risk of microcephaly and other CNS disorders in an infant born to a Zika-infected mother from 0.95% based on a study in French Polynesia up to 19.05% based on a recent study in Brazil.[18, 19] Other key parameters explored were the direct medical costs of microcephaly and other CNS disorders, the ratio of symptomatic to asymptomatic Zika infection, pregnant patient participation in Zika, microcephaly and other CNS disorder screening, and the hours of productivity lost from different types of care sought (primary versus specialist care) for symptomatic Zika infections. We report statewide cost estimates (given the range of vectors) and the cost per Zika case, which can be extrapolated to estimate costs for any number of Zika cases (e.g., specific outbreak size, town, city, etc.).

## Results

The model covers a Zika epidemic that spans 230 days—a duration determined by fitting the Zika-related microcephaly outbreak in Northeast Brazil[8] using case data up to June 4, 2016.[31] The results correspond to ranging attack rates from 0.01% up to 10% across the six states. The figures show attack rates from 0.01% to 1% to highlight the variability in increasing costs at these lower rates, while other results show the full range up to 10%.

### Clinical outcomes

The key clinical outcomes vary by attack rate for the six states (Table 2). For the base case in the six states at an attack rate of 0.05%, we estimate there will be 6,470 symptomatic infections, 21 cases of GBS, and 10 children with microcephaly. An attack rate of 0.10%, would result in 12,941 symptomatic infections, 42 cases of GBS, and 20 cases of microcephaly, while at an attack rate of 0.75%, we estimate 97,056 symptomatic infections, 317 GBS cases, and 150 microcephaly cases. Because the number of cases is dependent on the attack rate and the population, at the same attack rate states with larger populations would experience a higher burden. In Texas, the state with the largest population in the Gulf Coast, an attack rate of 0.05% would result in 2,522 symptomatic cases, 8 GBS cases, and 4 cases of microcephaly and other CNS disorders, whereas 0.10% would result in 5,044, 16, and 9, and 0.75% in 37,828, 124, and 66. For every 2-fold increase in attack rate, the number of GBS cases increases 2-fold. For example, at an attack rate of 0.25%, the number of expected GBS cases is 106 and at an attack rate of 0.5% the number of expected GBS cases is 211 (Table 2).

**Table 2:** Potential number of cases and costs for specific attack rates.

Outcome	Total	Alabama	Florida	Georgia	Louisiana	Mississippi	Texas
Attack rate = 0.01%							
Cases							
Infections	7,047	486	2,027	1,021	467	299	2,747
Microcephaly and other CNS	2 (1–11)	0 (0–1)	0 (0–3)	0 (0–2)	0 (0–1)	0 (0–0)	1 (0–5)
GBS	4 (1–6)	0 (0–0)	1 (0–2)	1 (0–1)	0 (0–0)	0 (0–0)	2 (1–2)
Cost							
Total	183 (58–439)	12 (4–28)	44 (14–106)	26 (8–63)	13 (4–31)	8 (2–18)	80 (25–193)
Productivity loss	66 (17–215)	4 (1–14)	16 (4–52)	9 (2–31)	5 (1–15)	3 (1–9)	29 (7–94)
Direct medical	117 (41–224)	8 (3–14)	28 (10–54)	17 (6–32)	8 (3–16)	5 (2–9)	51 (18–99)
Medicaid	22 (8–42)	2 (1–3)	6 (2–11)	3 (1–6)	3 (1–5)	1 (0–2)	8 (3–15)
Attack rate = 0.075%							
Cases							
Infections	52,857	3,644	15,203	7,661	3,503	2,244	20,602
Microcephaly and other CNS	15 (4–83)	1 (0–5)	4 (1–20)	2 (1–12)	1 (0–6)	1 (0–3)	7 (2–37)
GBS	32 (11–42)	2 (1–3)	9 (3–12)	5 (2–6)	2 (1–3)	1 (0–2)	12 (4–16)
Cost							
Total	249 (76–757)	16 (5–49)	61 (19–184)	36 (11–108)	17 (5–53)	10 (3–32)	109 (33–331)
Productivity loss	122 (32–485)	8 (2–31)	30 (8–118)	18 (5–69)	9 (2–34)	5 (1–20)	53 (14–212)
Direct medical	127 (43–272)	8 (3–17)	31 (11–66)	18 (6–39)	9 (3–19)	5 (2–11)	56 (19–119)
Medicaid	24 (8–51)	2 (1–4)	6 (2–13)	3 (1–7)	3 (1–6)	1 (0–3)	3 (1–5)
Attack rate = 0.25%							
Cases							
Infections	176,193	12,147	50,678	25,537	11,677	7,481	68,673
Microcephaly and other CNS	50 (14–278)	3 (1–18)	12 (3–67)	7 (2–40)	4 (1–19)	2 (1–12)	22 (6–122)
GBS	106 (35–141)	7 (2–10)	30 (10–41)	15 (5–20)	7 (2–9)	4 (1–6)	41 (14–55)
Cost							
Total	427 (125–1,613)	28 (8–104)	105 (31–394)	61 (18–231)	30 (9–112)	18 (5–67)	185 (54–705)
Productivity loss	273 (75–1,214)	18 (5–78)	68 (19–296)	39 (11–174)	19 (5–85)	11 (3–51)	118 (32–530)
Direct medical	154 (51–399)	10 (3–26)	38 (23–97)	22 (7–57)	11 (4–28)	6 (2–17)	67 (22–175)
Medicaid	29 (10–75)	2 (1–6)	7 (2–19)	4 (1–10)	3 (1–9)	2 (1–4)	10 (3–27)
Attack rate = 0.5%							
Cases							
Infections	352,387	24,295	101,356	51,074	23,354	14,962	137,346
Microcephaly and other CNS	100 (28–556)	6 (2–36)	24 (7–135)	14 (4–80)	7 (2–39)	4 (1–23)	44 (12–245)
GBS	211 (70–282)	15 (5–19)	61 (20–81)	31 (10–41)	14 (5–19)	9 (3–12)	82 (27–110)
Cost							
Total	680 (196–2,836)	44 (13–183)	169 (49–693)	97 (28–406)	47 (14–198)	28 (8–119)	294 (85–1,239)
Productivity loss	488 (135–2,255)	32 (9–145)	122 (34–551)	70 (19–322)	34 (9–157)	20 (6–94)	211 (58–985)
Direct medical	192 (61–581)	12 (4–37)	47 (15–142)	27 (9–83)	13 (4–41)	8 (3–24)	83 (27–254)
Medicaid	36 (11–109)	3 (1–8)	9 (3–28)	5 (2–15)	4 (1–12)	2 (1–6)	13 (4–40)
Attack rate = 1%							
Cases							
Infections	704,773	48,590	202,713	102,149	46,707	29,923	274,691
Microcephaly and other CNS	200 (55–1,113)	13 (4–71)	48 (13–269)	29 (8–159)	14 (4–78)	8 (2–46)	88 (24–489)
GBS	423 (141–564)	29 (10–39)	122 (41–162)	61 (20–82)	28 (9–37)	18 (6–24)	165 (55–220)
Cost							
Total	1,187 (336–5,283)	77 (22–341)	296 (84–1,292)	170 (48–755)	82 (23–368)	50 (14–221)	512 (145–2,306)
Productivity loss	919 (255–4,337)	60 (17–280)	229 (64–1,060)	132 (37–620)	64 (18–302)	38 (11–181)	396 (110–1,893)
Direct medical	268 (82–946)	17 (5–61)	66 (20–232)	38 (12–135)	19 (6–66)	11 (3–40)	116 (35–412)
Medicaid	50 (15–178)	4 (1–13)	13 (4–45)	7 (2–25)	6 (2–20)	3 (1–10)	18 (6–64)
Attack rate = 2%							
Cases							
Infections	1,409,545	97,180	405,425	204,297	93,414	59,847	549,382
Microcephaly and other CNS	401 (111–2,226)	26 (7–143)	97 (27–538)	57 (16–318)	28 (8–156)	17 (5–93)	176 (49–978)
GBS	846 (282–1,128)	58 (19–78)	243 (81–324)	123 (41–163)	56 (19–75)	36 (12–48)	330 (110–440)
Cost							
Total	2,201 (618–10,175)	143 (40–656)	550 (154–2,489)	315 (89–1,455)	152 (43–709)	92 (26–425)	949 (266–4,440)
Productivity loss	1,781 (496–8,501)	116 (32–548)	445 (124–2,078)	255 (71–1,216)	123 (34–592)	75 (21–355)	767 (213–3,711)
Direct medical	420 (122–1,674)	27 (8–108)	105 (30–411)	60 (17–239)	29 (8–117)	18 (5–70)	182 (53–729)
Medicaid	79 (23–314)	6 (2–23)	20 (6–80)	11 (3–44)	9 (3–36)	4 (1–17)	28 (8–114)
Attack rate = 5%							
Cases							
Infections	3,523,865	242,949	1,013,564	510,743	233,536	149,617	1,373,456
Microcephaly and other CNS	1,002 (277–5,564)	64 (18–357)	242 (67–1345)	143 (40–795)	70 (19–389)	42 (12–232)	440 (122–2,445)
GBS	2,114 (705–2,819)	146 (49–194)	608 (203–811)	306 (102–409)	140 (47–187)	90 (30–120)	824 (275–1,099)
Cost							
Total	5,243 (1,462–24,853)	341 (95–1,604)	1,311 (366–6,081)	751 (210–3,555)	363 (101–1,731)	220 (61–1,039)	2,257 (629–10,843)
Productivity loss	4,365 (1,219–20,993)	284 (79–1,354)	1,091 (305–5,133)	625 (175–3,002)	302 (84–1,463)	183 (51–878)	1,879 (524–9,163)
Direct medical	878 (244–3,860)	57 (16–249)	220 (61–948)	126 (35–552)	61 (17–269)	37 (10–162)	378 (105–1,680)
Medicaid	165 (46–729)	12 (3–54)	43 (12–186)	23 (6–101)	19 (5–83)	9 (3–40)	59 (16–262)
Attack rate = 10%							
Cases							
Infections	7,047,727	485,898	2,027,127	1,021,486	467,072	299,233	2,746,911
Microcephaly and other CNS	2,004 (555–11,128)	129 (36–715)	485 (134–2,691)	286 (79–1,590)	140 (39–778)	84 (23–465)	880 (244–4890)
GBS	4,229 (1,410–5,638)	292 (97–389)	1,216 (405–1,622)	613 (204–817)	280 (93–374)	180 (60–239)	1,648 (549–2,198)
Cost							
Total	10,313 (2,801–49,315)	672 (187–3,183)	2,584 (719–12,074)	1,480 (411–7,056)	715 (199–3,437)	433 (120–2,062)	4,444 (1,235–21,524)
Productivity loss	8,673 (2,423–41,812)	564 (158–2,697)	2,169 (607–10,225)	1,243 (347–5,980)	600 (168–2,913)	363 (101–1,748)	3,733 (1,042–18,249)
Direct medical	1,654 (448–7,524)	108 (29–486)	415 (112–1,849)	237 (64–1,076)	114 (31–524)	69 (19–315)	711 (193–3,274)
Medicaid	311 (84–1,413)	23 (6–105)	81 (22–362)	43 (12–196)	35 (10–161)	17 (5–78)	111 (30–511)

This table shows results for specific attack rates across the six states and combined. The costs are in millions of 2016 United States dollars [estimate (range: more conservative-less conservative)]; CNS = central nervous system disorders. GBS = Guillain-Barré Syndrome.

### Medical resource utilization

Of relevance to local, state, and federal health care system planners is the Zika-related increase in medical resource utilization. We estimated that an attack rate of 0.01% would produce 770 outpatient visits for Zika-infected non-pregnant persons, 350,464 ultrasounds for pregnant women, and 1 amniocentesis for pregnant women, whereas for attack rates of 0.025%, 0.5%, 2% and 10% would result in 1925, 350,411, and 3; 38,501, 348,7464, and 60; 154,002, 343,489, and 240; and 770,011, 315,449, and 1202, respectively. Under the base case assumption where 60% of pregnant women seek additional screening for infection (screening compliance), each additional 1% increase in attack rate would translate into 3,505 additional screening visits.

### Direct medical costs

**Fig 2:**
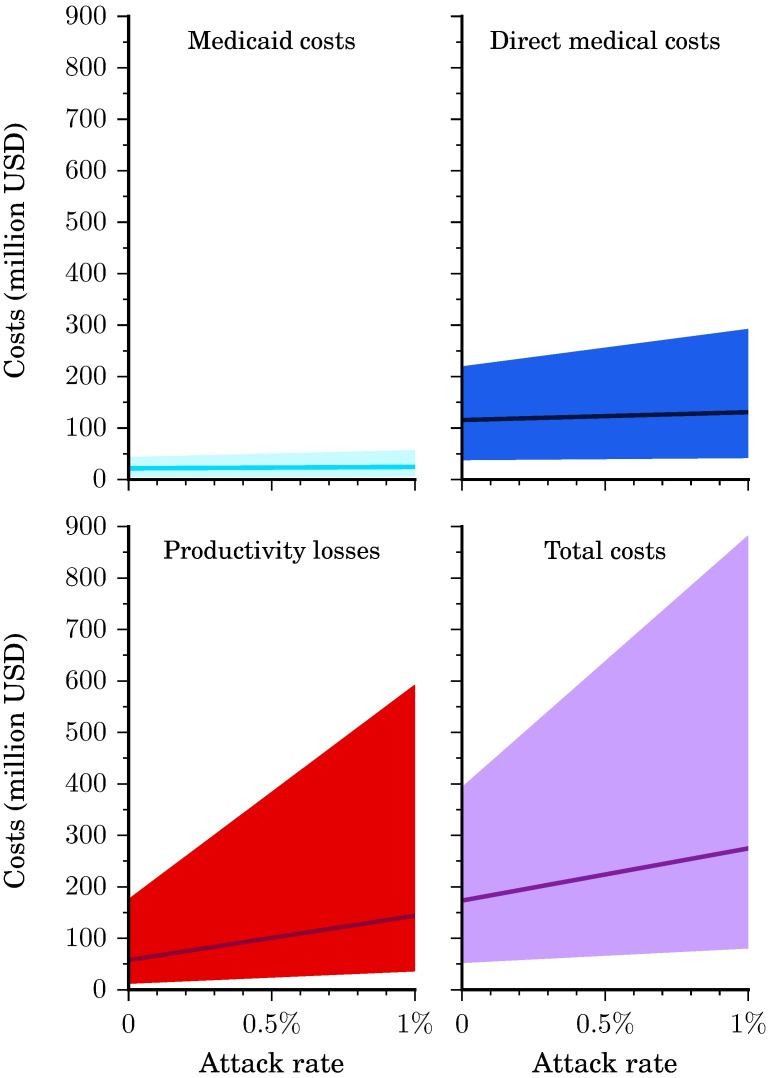
Total costs for all states combined. Medicaid costs, direct medical costs, productivity losses, and total costs for all six states combined. Illustrated here is the base case scenario (solid line) as well as the range from the more conservative to the less conservative scenario (shaded region).

Direct medical costs remained relatively insensitive to attack rates for all states, (Table 2) as the majority of medical costs were due to additional screenings and ultrasounds in uninfected pregnant women. However, for an attack rate larger than 0.10%, the increasing incidence of symptomatic Zika infections, GBS cases, and microcephaly/CNS disorders was the main component in the direct medical costs. For example, we estimated that an attack rate of 0.05% across all six states would result in $123.2 million ($42.5-$242.4 million) direct medical costs, while an attack rate of 0.10% would result in of $130.8 million ($44.5-$289.8 million), and an attack rate of 0.75% in $229.9 million ($71.0-$763.4 million) direct medical costs (Fig 2).

Direct medical costs vary by the population at risk and the attack rate (Table 3). Symptomatic cases excluding pregnant women would result in costs of $1.9 million ($0.26-$6.5 million) for an attack rate of 0.25% and $15.5 million ($2.1-$52.0 million) for an attack rate of 2%. Costs for pregnant, non-infected women remain relatively stable because the number of additional screenings does not change substantially. Cases of microcephaly and other CNS disorders and GBS increase dramatically as the attack rate increases.

**Table 3:** Direct medical costs by at risk group for all states combined.

Attack rate	Symptomatic, infected, non-pregnant, non-complicated	Pregnant, non-infected	Pregnant, infected, asymptomatic, normal US)	Pregnant, infected, symptomatic, normal US)	Pregnant, infected, abnormal US	Microcephaly and other CNS	GBS
0.01%	0.08 (0.01–0.26)	116 (40–217)	0.02 (0.004–0.03)	0.005 (0.0006–0.01)	0.002 (0.0004–0.02)	1 (0.33–7)	0.24 (0.07–0.38)
0.075%	0.58 (0.08–2)	115 (40–217)	0.16 (0.03–0.24)	0.04 (0.005–0.10)	0.01 (0.003–0.15)	9 (2–50)	2 (0.50–3)
0.25%	2 (0.26–6)	115 (40–216)	0.52 (0.09–0.82)	0.13 (0.16–0.32)	0.05 (0.01–0.50)	30 (8–165)	6 (2–9)
0.5%	4 (0.52–13)	115 (40–216)	1 (0.18–2)	0.26 (0.03–0.64)	0.09 (0.02–1)	59 (16–330)	12 (3–19)
1%	8 (1–26)	114 (40–215)	2 (0.36–3)	0.52 (0.06–1)	0.18 (0.04–2)	119 (33–660)	24 (7–38)
2%	16 (2–52)	113 (40–213)	4 (0.72–7)	1 (0.13–3)	0.37 (0.08–4)	238 (66–1,321)	48 (13–76)
5%	39 (5–130)	110 (38–206)	10 (2–16)	3 (0.32–6)	0.92 (0.19–10)	595 (165–3,302)	121 (33–189)
10%	78 (10–260)	104 (36–195)	21 (4–33)	5 (0.65–13)	2 (0.38–20)	1,189 (329–6,605)	241 (67–378)

This table shows direct medical cost results for specific attack rates across by various at risk groups for all states combined. The costs are in millions of 2016 United States dollars [estimate (range: more conservative-less conservative)]; US = ultrasound. CNS = central nervous system disorders. GBS = Guillain-Barré Syndrome.

**Fig 3:**
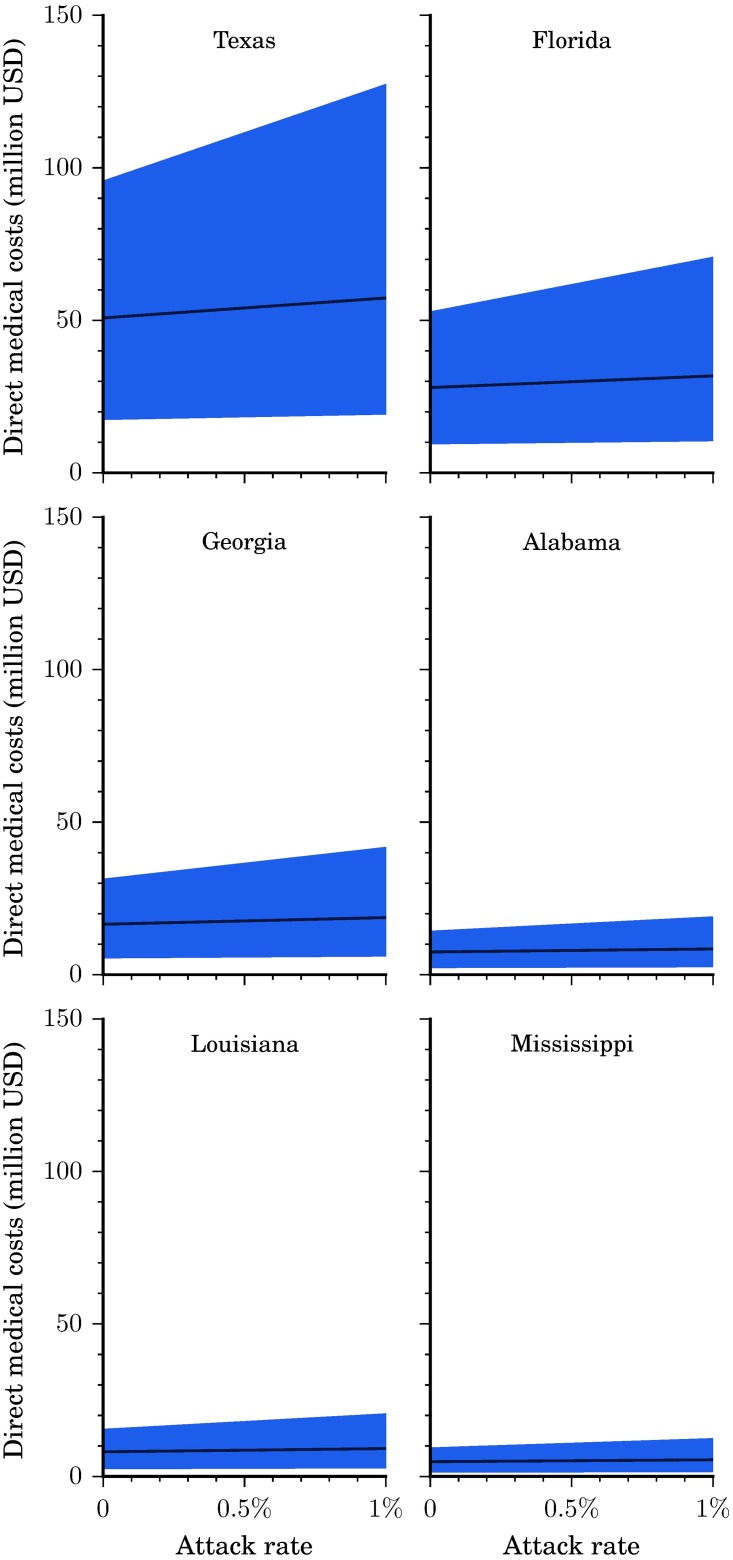
Direct medical costs per state. Direct medical costs by state and attack rate. Illustrated here is the base case scenario (solid line) as well as the range from the more conservative to the less conservative scenario (shaded region).

Because of different population sizes and birth rates, each state would be expected to incur very different total costs as well as costs per case, at the same attack rate. For example, at attack rates of 0.05% and 0.75%, Mississippi would incur direct medical costs of $5.1 million ($1.8-$10.6 million) and $9.6 million ($3.0-$31.9 million), respectively, while Texas would incur direct medical costs of $54.0 million ($18.7-$100.2 million) and $99.8 million ($30.9-$333.1 million), respectively (Fig 3).

### Medicaid costs

**Fig 4:**
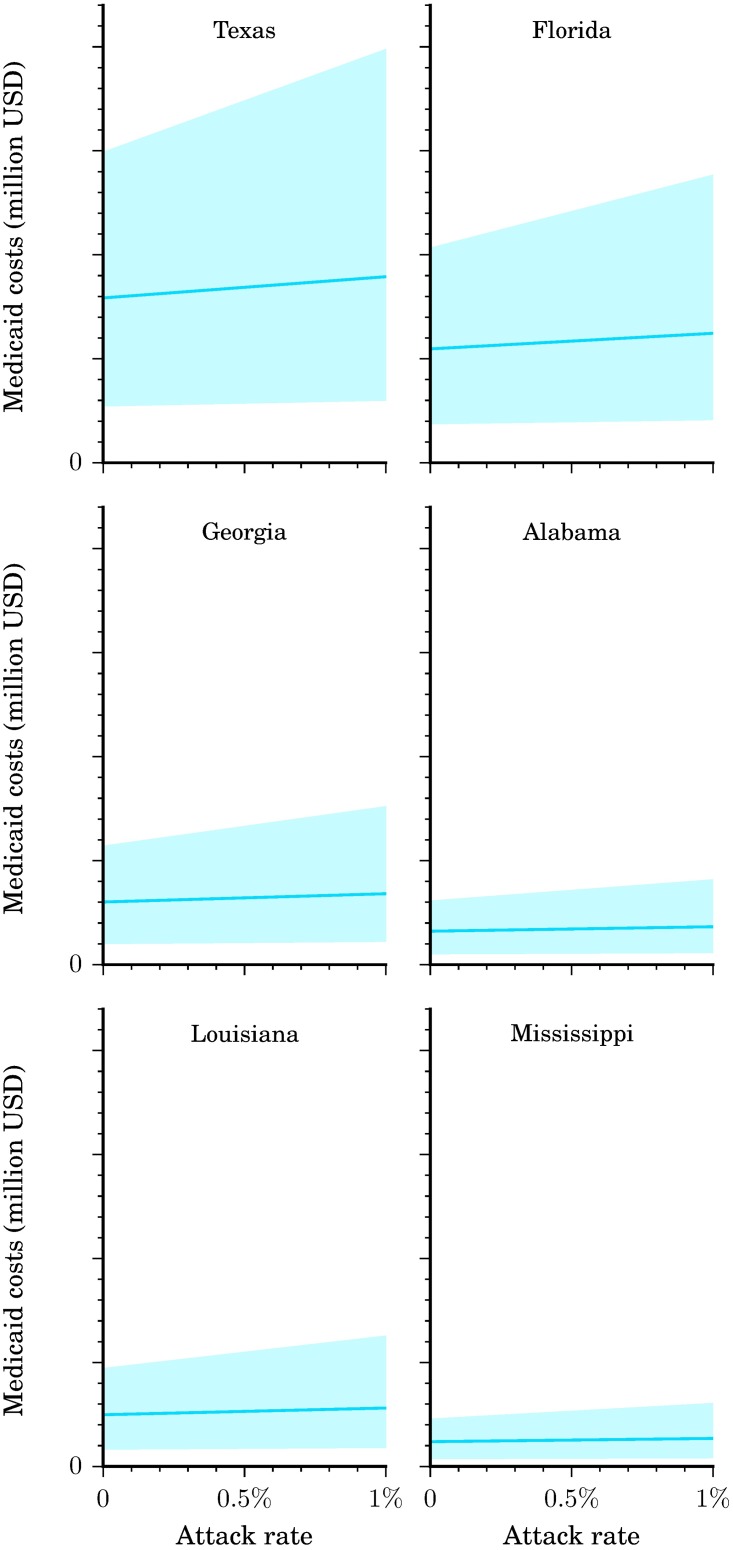
Medicaid costs per state. Medicaid costs by state and attack rate. Illustrated here is the base case scenario (solid line) as well as the range from the more conservative to the less conservative scenario (shaded region).

Because Medicaid costs are a subset of direct medical costs, they are also relatively insensitive to variations in the attack rate (Fig 4). At low attack rates (i.e., <1%), total Medicaid costs consisted largely of additional screenings and ultrasounds in uninfected, pregnant women. As attack rates increased, a greater percentage of total Medicaid costs was due to additional outpatient visits for symptomatic infections and the long-term costs of GBS and CNS disorders. For example, in Louisiana, an attack rate of 0.05% resulted in Medicaid costs of $2.6 million ($0.9-$5.4 million), 0.10% in $2.8 million ($1.0-$6.2 million), and 0.75% in $4.9 million ($1.5-$16.4 million) (Fig 4). Of the six states, Louisiana incurred the highest Medicaid cost per Zika infection: $1,134 ($391-$2,332) at a 0.05% attack rate, $601 ($205–1,333) at 0.10%, and $140 ($43-$467) at 0.75%.

### Productivity losses

**Fig 5:**
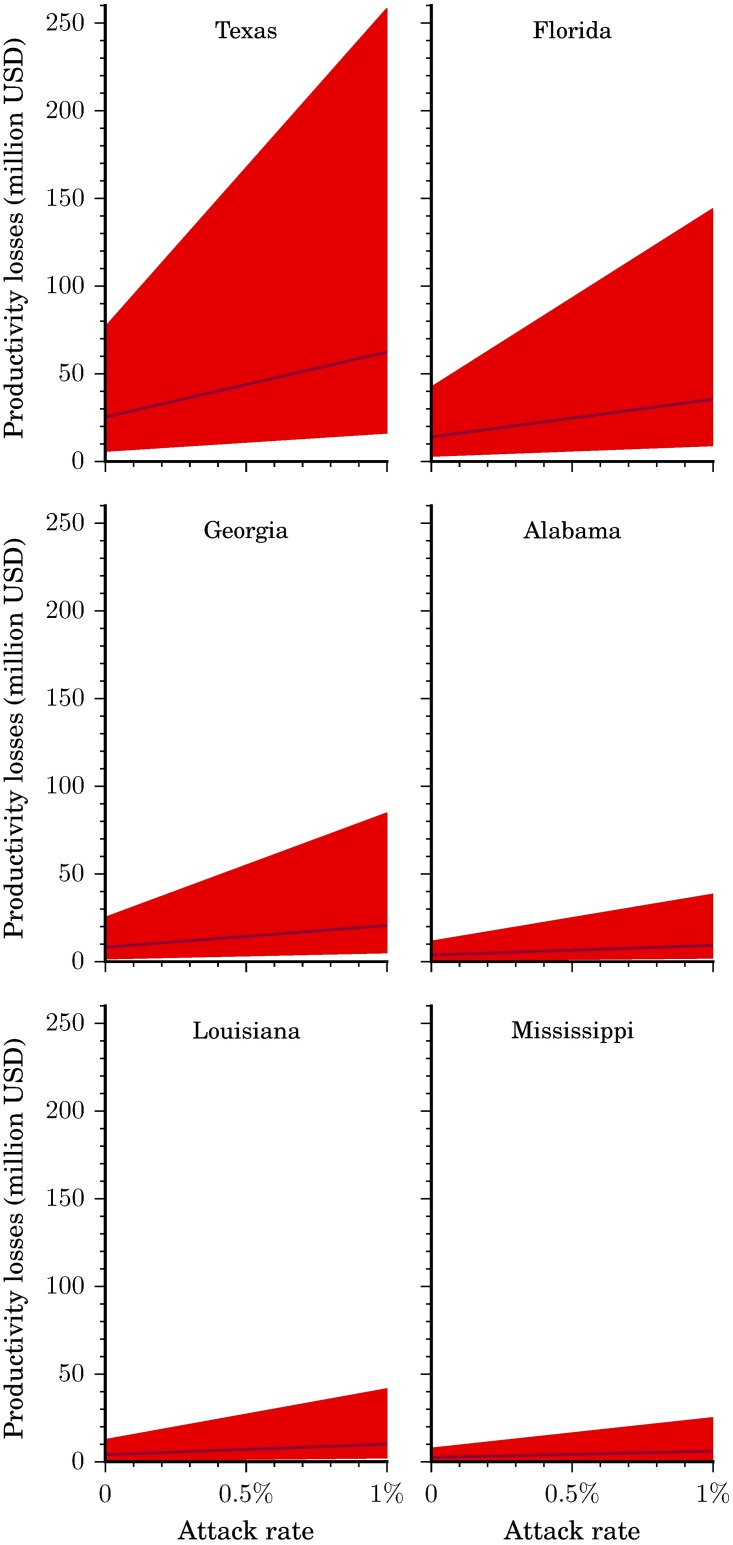
Productivity losses per state. Total productivity losses by state and attack rate. Illustrated here is the base case scenario (solid line) as well as the range from the more conservative to the less conservative scenario (shaded region).

As can be seen in Table 2, productivity losses comprised a majority of the Zika outbreak-related costs with well over 50% of costs stemming from time taken for additional screenings and ultrasounds in pregnant women and the lifetime productivity losses for persons with GBS and CNS disorders (Fig 5). Total productivity losses were $100.8 million ($26.5-$316.4 million) at an attack rate of 0.05% and $703.8 million ($195.1 million-$3.3 billion) at an attack rate of 0.75% (Fig 2). Productivity losses were attenuated on average by 45% (43–46%) across the states when analyzing only the population employed. At an attack rate of 0.01%, productivity losses were $35 million ($9-$121 million) for all states, down from $66 million ($17-$215 million) when the entire population was represented.

### Total costs

**Fig 6:**
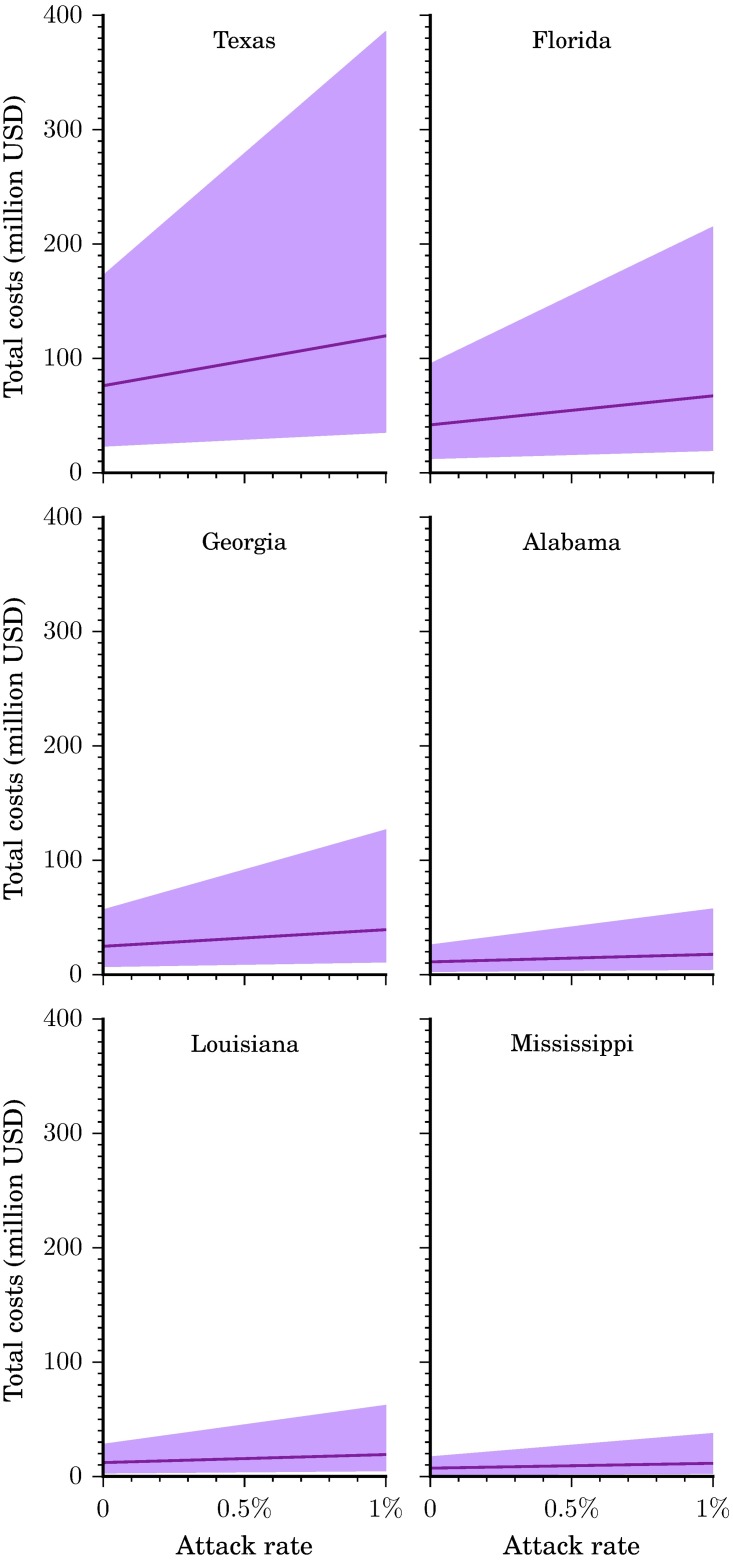
Total costs per state. Total costs (direct medical and productivity losses) by state and attack rate. Illustrated here is the base case scenario (solid line) as well as the range from the more conservative to the less conservative scenario (shaded region).

Total costs are comprised of direct medical costs plus productivity losses (Figs 2 and 6). We estimated that an attack rate of 0.05% in all six states would yield a total cost of $223.9 million ($68.9-$558.8 million) or $6,355 ($1,956-$15,858) per Zika infection, whereas an attack rate of 0.1% would yield a total cost of $274.6 million ($83.0-$879.3 million) or $3,897 ($1,178-$12,476) per infection, and one of 0.75% would yield a total cost of $933.7 million ($266.0 million-$4.1 billion) or $1,767 ($503-$7,680) per infection. Assuming the base case scenario, at an attack rate of 0.05%, the cost per infection would be $5,939 in Alabama, $5,385 in Florida, $6,270 in Georgia, $6,688 in Louisiana, $6,256 in Mississippi, and $7,130 in Texas would per infection. An attack rate of 0.1% would be $3,648 in Alabama, $3,319 in Florida, $3,846 in Georgia, $4,095 in Louisiana, $3,838 in Mississippi, and $4,359 in Texas per infection and at 0.75% would be $1,664 in Alabama, $1,528 in Florida, $1,745 in Georgia, $1,848 in Louisiana, $1,742 in Mississippi, and $1,958 in Texas per infection. These costs per infection can be extrapolated to estimate the cost for any number of cases. For example, 100 cases that occur in Texas could cost an average of $161,571 to $2.9 million for attack rates ranging from 10% to 0.01%.

## Discussion

Given the tremendous uncertainty surrounding potential attack rates for Zika in the US, our model and study demonstrated how various outcomes and costs would vary based on attack rate and other circumstances. Our study identified the thresholds at which the economic impact of Zika on the US Gulf Coast would exceed $0.5, $1, $1.5, and $2 billion. For example, in the base case scenario, an attack rate of approximately 0.3% would exceed $0.5 billion, an attack rate of 1% would exceed $1 billion, an attack rate of approximately 1.3% would exceed $1.5 billion, and an attack rate of 2% would exceed $2 billion in total costs.

Without details regarding the Zika-prevention measures that would be implemented and how effective these may be, it is unclear what percentage of these costs may be averted. Possible targets of Zika control investment could be improved vector control, more extensive Zika screening, and accelerated vaccine development. Assuming a third of the costs could be averted, an attack rate of 1% would justify investments in the $1 billion range. Such an attack rate would be substantially lower than those observed in French Polynesia (66%, 95% confidence interval (CI) 62–70%),[19] Yap Island, Micronesia (73%, 95% CI 68–77%),[3] as well as the attack rates for recent outbreaks of chikungunya, a flavivirus with the same vector, including the Puerto Rico outbreak (23.5%).[32]

Since the US lacks a comprehensive surveillance system for Zika, no one knows the exact geographic spread of this novel endemic to date, requiring us to rely on assumptions and hypothetical scenarios. As both Zika and dengue are transmitted by the same mosquito,[29] one possibility is that Zika’s spread may be similar to that of dengue. The last dengue outbreaks in the US occurred in Hawaii in 2001, in Texas in 2005,[33, 34] and in Florida in 2010. Despite the meteorological suitability for the vectors during the summer months,[29] roughly 100 cases of dengue are reported annually in the US.[34] While most cases are travel-related, autochthonus transmission of dengue in the US is becoming increasingly possible.[34] The Zika virus does have some important distinctions from dengue, such as additional routes of transmission like sexual, perinatal, and possibly transfusion,[35, 36] and the population’s lack of exposure and thus immunity to the virus (since Zika is a novel virus compared to dengue) that could further facilitate its spread. Therefore, our study used the assumptions outlined in our previously published modeling study.[8]

The economic impact of infectious diseases is not always readily apparent and may be significantly underestimated, especially when manifested in life-long clinical outcomes. Since Zika may not pose an appreciable risk of mortality to infected adults, it is possible to overlook for the full extent of its impact. The most publicized effect of Zika has been the risk of microcephaly to children born to Zika-infected pregnant women. The costs of microcephaly are borne out over decades and thus may be difficult to evaluate without quantitative analyses. Our understanding of Zika is rapidly evolving as it becomes apparent that its clinical outcomes include not only microcephaly, but an array of other congenital impairments, such as vision-threatening ocular lesions.[37] In addition, our analyses demonstrated that the cumulative costs of uncomplicated, yet symptomatic Zika infection, are also considerable. While the costs associated with an uncomplicated Zika infection may seem minimal on an individual level (e.g., an outpatient visit and lost productivity of no more than one day), these costs can accumulate across the population. As has been demonstrated with other diseases, such as influenza, important components of disease costs are those associated with “milder” clinical outcomes, irrespective of hospitalization and death. When such outcomes are prevalent, they can have an impact on employers as well as society as a whole.[38] Our study shows how sensitive the societal economic burden is to the attack rate of Zika, which underscores the value of early containment.

As we aimed to be conservative in our estimations, our model in many ways may underestimate the economic burden of Zika. Since there is a dearth of previous estimates of the economic impact of microcephaly, we used the costs associated with autism as a proxy. However, the costs of autism are likely to be lower than those of microcephaly. Microcephaly can result in a number of clinical sequelae such as significant sensory and motor impairment that would require greater medical and personal care than most cases of autism. Furthermore, many cases of microcephaly and other CNS disorders may be difficult to diagnose until infants fail to reach milestones; weeks or months after birth. These initially undiagnosed cases will have substantial health burdens, for which the true cost is not yet incorporated into our calculations. In addition, we did not account for the potential lost productivity and medical costs associated with stress due to fear of Zika and concern over family and friends suffering sequelae of Zika infection, as demonstrated by other studies on previous epidemics such as H1N1[39] and Ebola.[40] Concerns about Zika can also disrupt economic activities, tourism and travel. Additionally, our study did not consider impact beyond the five Gulf Coast States and Georgia, although several mechanisms, including sexual transmission and travel, could lead to Zika cases occurring elsewhere in the US.

### Limitations

The goal of this study was not to predict the course of the Zika epidemic, but to delineate how the economic burden may vary under different attack rates and circumstances. By definition models are simplifications that aim to distil systems down to the most pertinent relationships and key factors without including extraneous detail. While our data-driven analyses drew from a variety of sources and locations, the current literature on Zika is limited and new data continues to emerge. For instance, there are wide discrepancies on the reported risk of microcephaly in infants born to Zika-infected mothers. Therefore, we performed sensitivity analyses to determine how such uncertainty may affect results and to identify needs for future data collection. Additionally, we used laboratory testing costs from CMS reimbursements, which may differ state to state. Third-party payers typically have financial agreements with medical providers for a different fee for each medical charge and can be different from Medicaid reimbursement. Therefore, our Medicaid costs are an approximation due to data restrictions. Awareness of the risk for Zika virus may affect the behavior of pregnant women and couples trying to conceive. However, we did not include any costs associated with the termination of pregnancy (either spontaneous or planned) as the likelihood of this due to Zika is unknown or not well established. This analysis focused on six states in the continental United States at high-risk of Zika infection. While Zika infections may be expected outside of this area, our model chose to conservatively focus on these regions. In addition, our model focuses on attack rates that are distributed across the state and are varied across a wide range to account for the potential impact of vector control activities. With this in mind, the entire population of a state, including pregnant women, falls under the at risk definition provided by the CDC.[10] However, as Zika continues to spread beyond the current regions, more of the state could become high-risk regions.

### Conclusions

To inform Zika funding options currently under review, our team developed a computational model to forecast the potential economic burden of Zika under different scenarios and circumstances and the circumstances under which these costs would exceed different thresholds. Our analyses indicate that the health and economic burden of even low attack rates of Zika in the Continental US would be both substantial and enduring.
